# Awareness about a Life-Threatening Condition: Ectopic Pregnancy in a Network for Surveillance of Severe Maternal Morbidity in Brazil

**DOI:** 10.1155/2014/965724

**Published:** 2014-03-19

**Authors:** Edilberto Alves Rocha Filho, Danielly Scaranello Santana, Jose Guilherme Cecatti, Maria Laura Costa, Samira Maerrawe Haddad, Mary Angela Parpinelli, Maria Helena Sousa, Rodrigo Soares Camargo, Rodolfo Carvalho Pacagnella, Fernanda Garanhani Surita, Joao Luiz Pinto e Silva

**Affiliations:** ^1^Department of Obstetrics and Gynecology, School of Medicine, State University of Campinas, R. Alexander Fleming 101, 13083-881 Campinas, SP, Brazil; ^2^Campinas Center for Studies in Reproductive Health (CEMICAMP), R. Vital Brasil 200, 13083-888 Campinas, SP, Brazil

## Abstract

*Objective.* To assess occurrence of severe maternal complications associated with ectopic pregnancy (EP). *Method.* A multicenter cross-sectional study was conducted, with prospective surveillance of potentially life-threatening conditions (PLTC), maternal near miss (MNM), and maternal death (MD). EP complications, patient sociodemographic/obstetric characteristics, and conditions of severity management were assessed, estimating prevalence ratios with respective 95% CI. Factors independently associated with greater severity were identified using multiple regression analysis. *Results.* Of the 9.555 severe maternal morbidity patients, 312 women (3.3%) had complications after EP: 286 (91.7%) PLTC, 25 (8.0%) MNM, and 1 (0.3%) MD. Severe maternal outcome ratio (SMOR) was 0.3/1000 LB among EP cases and 10.8/1000 LB among other causes. Complicated EP patients faced a higher risk of blood transfusion, laparotomy, and lower risk of ICU admission and prolonged hospitalization than women developing complications resulting from other causes. Substandard care was the most common in more severe maternal morbidity and EP cases (22.7% MNM and MD versus 15% PLTC), although not significant. *Conclusion.* Increased maternal morbidity due to EP raised awareness about the condition and its impact on female reproductive life. No important risk factors for greater severity were identified. Care providers should develop specific guidelines and interventions to prevent severe maternal morbidity.

## 1. Introduction

Ectopic pregnancy (EP) is a recurrent medical condition. It is a significant cause of maternal mortality and morbidity, especially in low-income and middle-income countries, where the majority of patients present late with tubal rupture and hemodynamic compromise [1]. The incidence of EP is approximately 1-2% of pregnancies and 10–20 per 1,000 live births [2, 3]. The overall incidence is currently rising worldwide, possibly due to increasing pelvic inflammatory disease (PID), with persistent luminal damage [4–8].

Despite the recently observed decline in general and specific maternal mortality due to ectopic pregnancy, this remains the cause of around 4.9% of all maternal deaths in developed countries, with 3-4% in the United States and in England [5, 6, 9, 10]. In low- and middle-income countries, it is estimated that approximately 0.5% of maternal deaths are due to ectopic pregnancy, with 0.5% in Latin America, 0.5% in Africa, and 0.1% in Asia [9, 11, 12]. Ectopic pregnancy is the main cause of maternal mortality in the first trimester of pregnancy and is responsible for 80% of maternal deaths in this phase, at least in settings where there are no restrictive laws for induced abortion [2, 5, 13].

Awareness about the impact of this condition on young fertile women and care providers is paramount, since early diagnosis can avoid severe intra-abdominal hemorrhage in tubal EP. The prevention of maternal morbidity in EP represents a major challenge to ensure improvement in women's health, since there are no identified risk factors that can clearly predict severe bleeding in these cases [3, 8].

As a secondary analysis from a multicenter cross-sectional study for the surveillance of severe maternal morbidity and near miss in Brazil [14], the aim of this study is to evaluate EP considering an innovative approach, using the WHO concepts [15] of potentially life-threatening condition (PLTC), maternal near miss (MNM), and maternal death (MD). Diagnostic criteria for the identification of the above-mentioned conditions, as well as the specific sociodemographic and obstetric characteristics associated with a worse outcome of EP, will be explored. Management criteria will also be assessed.

## 2. Materials and Methods

A multicenter cross-sectional study was conducted in 27 referral obstetric units in diverse geographical regions in Brazil. During a 12-month period, from June 2009 to May 2010, prospective surveillance of potentially life-threatening maternal conditions (PLTC), maternal near miss (MNM), and maternal death (MD) was carried out, using the WHO criteria and classification [15–17]. Sample size was originally estimated by roughly 75.000 deliveries that should be under surveillance to identify near miss cases by using the new criterion established by the World Health Organization.

Thus, all medical charts of women admitted to participating hospitals to deliver or because they have any severe complication related do pregnancy were reviewed immediately after hospital discharge. Medical charts of women transferred to other healthcare services before completion of the case or those who died were also reviewed, in search of cases showing the WHO identifiers defined as those most frequently associated with organ dysfunction and severe morbidity. The search for information that was unavailable in the chart was carried out in other sources such as the hospital database, prenatal record forms, and transfer documents or was obtained from the healthcare team.

Data collection was conducted in a specific chart that also contained information about adequacy of health care and the occurrence of delays for getting appropriate treatment. After manual collection, the forms were filed to become accessible at the time of technical visits for quality control. The data was entered into electronic forms hosted on the project website, which was hosted on the institutional web page of the coordinating study center and sent to the central database, using the OpenClinica 3.0, a specific platform for management of clinical studies. Further details on the study and methodological aspects are included in other publications [14, 18]. Approval from the IRB of each institution and from the National Council for Ethics in Research was obtained before the beginning of the study.

Quality control was assured by several manners. Initially, before data collection began, a manual of operation was provided and coordinators of each center were trained. During data collection, each coordinator reviewed the forms, checked for typographical errors, and provided the search for data that was unavailable on the charts. The local investigator carried out a new review to identify possible inconsistencies. Finally, the national study coordinators reviewed the database, identified inconsistencies, and sent the correction report to the participating centers which were required to make the corrections [18].

During the study, consistent auditing with a set of validation and cross-checking rules as part of online data management assigned a systematic evaluation of possible delays and deficiencies in the quality of care and health system inadequacy, with data on interhospital transfer, refusal by a patient to accept treatment (“discharge requested by the patient” or “evasion”), or lack of equipment or medication. Altogether they are operationally defined as a substandard care with or without delays.

For the assessment of ectopic pregnancy associated with severe maternal morbidity, cases were divided into obstetric complications due to ectopic pregnancy and obstetric complications due to other causes. Therefore not all cases of ectopic pregnancy entered the study, but only those complicated with a specific life-threatening condition identified or those undergoing a laparotomy for treatment. Initially, the prevalence of PLTC, MNM, and MD was calculated and compared between these groups. Then the following health indicators related to maternal morbidity and mortality were estimated: the maternal near miss incidence ratio (MNM incidence ratio), severe maternal outcome ratio (SMOR, MNM + MD), maternal near miss to maternal death ratio (MNM : MD ratio), and mortality index and maternal mortality ratio (MMR), according to WHO recommendations [15].

The diagnostic criteria used for the identification of PLTC, MNM, and MD, as well as the conditions of severity management, were assessed in these same groups (ectopic pregnancy and other causes). The *P* value for diagnostic criteria and the prevalence ratio adjusted for cluster effect of the design with their respective 95% CI for the conditions of severity management were estimated. The correction for the cluster effect of the design was performed because each participating center was considered to be a cluster, and the correspondent heterogeneity of values within each variable among cluster was assessed as adequate [19]. With the purpose of evaluating the sociodemographic and obstetric factors possibly related to greater severity among women with complications secondary to ectopic pregnancy, two comparative groups were created: one with PLTC and the other with more severe conditions, represented by the sum of MNM and MD. Then, we calculated the prevalence ratio adjusted for cluster effect with the respective 95% CI. Finally, a multiple Poisson regression analysis was used to identify the factors independently associated with greater severity of complications due to ectopic pregnancy.

## 3. Results

**Figure 1:**
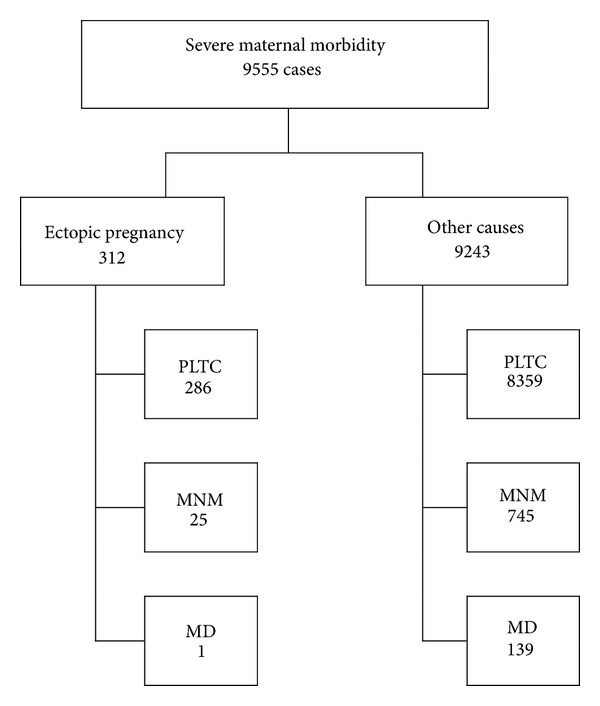
Flow of women with severe maternal morbidity due to ectopic pregnancy or other causes according to the final outcome in PLTC (potentially life-threatening condition), MNM (maternal near miss), or MD (maternal death).

In a total of 9.555 women identified with severe complications associated with pregnancy, delivery, or postpartum period, 312 (3.3%) had complications secondary to ectopic pregnancy and 9.243 (96.7%) developed complications resulting from other causes. PLTC and MNM, respectively, occurred in a total of 8.359 (90.4%) and 745 (8.1%) women in the group with other causes and in 286 (91.7%) and 25 (8.0%) women in the EP group. MD occurred in a total of 139 (1.5%) women for the morbidity group due to other causes. There was only one death (0.3%) attributed to ectopic pregnancy (Table 1) (Figure 1). This only one case of maternal death due to ectopic pregnancy was admitted to one of the participating centers already in an extreme severe, hemorrhagic shock, and almost dying condition after a laparotomy performed in another hospital. The women died soon after admission in the intensive care unit.

The maternal near miss incidence ratio was 0.3/1000 LB among ectopic pregnancy cases and 9.07/1000 LB among the remaining causes; the severe maternal outcome ratio (SMOR) was 0.3/1000 LB among ectopic pregnancy cases and 10.8/1000 LB among the remaining causes. The MNM : MD ratio was 25 : 1 for ectopic pregnancy cases and 5.4 : 1 for the remaining causes. The mortality index was 3.8% for ectopic pregnancy cases and 15.7% for the remaining causes and the maternal mortality ratio (MMR) was 1.2/100.000 LB among ectopic pregnancy cases and 169.2/100.000 LB among other causes (Table 1).

Bleeding was the most widely used diagnostic criteria for PLTC in the identification of complicated cases of ectopic pregnancy, while, among the remaining causes, hypertension and clinical-surgical criteria were more frequently used. Infection was not identified as statistically significant for morbidity due to EP, in comparison to the remaining causes (Table 2).

Among the more severe cases, maternal near miss and maternal death (MNM and MD), the most widely used criteria for complicated EP were clinical (16 MNM cases) and management (15 MNM cases), when applying the WHO criteria for identifying near miss events. In the only MD case, clinical, laboratory, and management criteria were observed (Table 2).

When assessing the conditions of severity management, it was observed that patients who had complicated ectopic pregnancy showed a higher estimated risk of blood product transfusion and laparotomy and a lower risk of ICU admission and prolonged hospitalization in comparison to patients presenting with complications secondary to other causes (Table 3).

The variables assessed on bivariate analysis (maternal age, marital status, school education, and skin color/ethnicity) did not show any significantly increased estimated risk of a worse prognosis (maternal near miss event or death) (Table 4). Concerning obstetric conditions, the occurrence of one or more prior abortions and the history of previous uterine surgery were identified as protective factors against the occurrence of severe complications secondary to ectopic pregnancy (Table 5). Substandard care was shown to be the most common among more severe cases of maternal morbidity due to EP, being identified in 22.7% of MNM and MD cases versus 15% in PLTC, but this was not a significant difference (Table 5).

Multivariate analysis (Table 6) identified that, among the factors evaluated simultaneously, the presence of previous uterine scar and nonwhite skin color was independently associated with protection against severe complications secondary to ectopic pregnancy.

## 4. Discussion

To the best of our knowledge, this is the first analysis of EP according to the new WHO concepts of potentially life-threatening condition, maternal near miss, and maternal death, in a large well-defined sample of women, with prospective nationwide data collection.

Much has been discussed about the risk factors for the occurrence of complications and death due to ectopic pregnancy, such as nonwhite skin color, presence of previous disorders (particularly diabetes mellitus), unstable marital status, lower level of school education, nulliparity, history of prior ectopic pregnancy, and delay in care provision [5, 10, 11]. However, specifically for near miss events and ectopic pregnancy, this assessment does not exist. Determining the risk for severe clinical course may help in the management of these women. Despite all laboratory and imaging advances that permit early diagnosis and treatment, ectopic pregnancy is still an important cause of maternal death [2, 5]. The WHO estimates that 4% of all maternal deaths occurring in developed countries and 0.5% in developing settings are due to ectopic pregnancy [9, 12]. In our study, we found 0.7% (1/140) of deaths related to ectopic pregnancy, a proportion lower than that observed in all developed countries. However, the number was close to the 0.5% observed in Latin America [9, 12]. Unfortunately, there are no comparative data for PLTC in the literature.

Specific mortality due to ectopic pregnancy alone does not fully describe obstetric care. Therefore, it is important to consider the health indicators described by the WHO in 2009 [15]. The maternal near miss incidence ratio and SMOR are aimed at estimating the complexity of care. Therefore, higher values meant that more women required high-complexity care. The MNM : MD ratio represents which proportion of near miss cases progressed to maternal death. The mortality index in turn represents an estimate of performance. Thus, when this index is high (higher than 20%), the quality of obstetric care provision for severe cases was not adequate [15, 17]. For the first time, these indicators recommended by the WHO have been described for ectopic pregnancy. Therefore, no results are available for comparison. Hopefully these figures will be worth for future comparisons with other population studies approaching maternal morbidity as the big one recently issued by the WHO [20].

The conditions of severe maternal morbidity related to ectopic pregnancy are associated with tubal rupture, rapid clinical deterioration due to major intra-abdominal bleeding, and posterior progression to hypovolemic shock, requiring blood transfusion [3, 10, 11]. Bleeding was actually the main diagnostic criteria used to identify PLTC cases in the current study. Furthermore, regarding conditions of severity management in patients with ectopic pregnancy, laparotomy and blood transfusion were the most important conditions. It is fundamental to consider that this study assessed a specific group of women with a severe condition. Cases in which an early diagnosis was made, allowing for clinical treatment with methotrexate or laparoscopic surgery, and unruptured EP in hemodynamically stable patients were not included in the current case study.

Another protective factor identified was history of prior abortion. According to Sindos et al., nulliparous patients tend to seek medical care earlier. As soon as these patients perceive any different symptoms such as pain or bleeding, they seek medical treatment and ectopic pregnancy is identified early, before tubal rupture [5]. The same association may be true for patients with a previous history of abortion [21]. Knowing the symptoms, these women would seek medical care sooner, which would allow an earlier diagnosis of ectopic pregnancy. This is a protective factor against the development of a more severe outcome associated with ectopic pregnancy. History of previous uterine surgery was also identified as a protective factor, possibly due to greater medical surveillance in these cases.

On multivariate analysis, apart from history of previous uterine surgery, we also found skin color as a protective factor, in contrast to descriptions in the literature reporting nonwhite skin color as a risk for the occurrence of complications and death from ectopic pregnancy. We should always bear in mind that the risk factors described are not related to near miss cases. There are still no comparative data for near miss cases in the literature and these cases may behave differently [5, 10, 11]. Furthermore, the widespread miscegenation of the Brazilian population might lead to some difficulty in clearly defining ethnicity.

One of the most interesting findings of the present study was information on the quality of care, with evidence of increased substandard care and/or delays in more severe cases. A similar suggestion was made by van Mello et al. in a case-control study, comparing EP patients developing complications after abdominal hemorrhage to hemodynamically stable EP patients. Those authors emphasized that since patient-related risk factors have not been consistent in identifying a worse outcome as yet, the key point would be to focus on awareness about EP and its clinical management [3]. Indirectly we could argue that the cases managed in places with better resources, easy access for women to health facility, and more trained health professionals had the ectopic pregnancy diagnosed earlier, before a severe complication developed, and these cases were managed clinically, perhaps with laparoscopy or with methotrexate. These cases were not enrolled at all in the current study because they are not classified as severe morbidity.

Some possible limitations of this study must be considered. As a secondary analysis of a larger study, information about important risk factors usually assessed for EP cases was lacking, for example, previous EP (data collection concerned previous history of abortion), history of pelvic inflammatory disease, and history of infertility. There was also a lack of data on diagnostic tools used for each case: ultrasound findings and hCG levels or clinical findings at diagnosis.

## 5. Conclusions

A relatively large number of maternal morbidity cases due to EP were found in the Brazilian population during the surveillance period, raising awareness of this condition and its impact on female reproductive life. No important risk factors for increased severity were identified. However, there seems to be deficient or substandard care associated with complicated EP cases. Further action taken would be to address care providers to develop specific guidelines and interventions for the prevention of severe maternal morbidity due to this specific condition.

## Figures and Tables

**Table 1:** Prevalence of potentially life-threatening conditions (PLTC), maternal near miss (MNM), and maternal deaths (MD) among complicated ectopic pregnancy cases and other causes of morbidity and their correspondent health indicators.

Morbidity/mortality	Cause		PR (95% CI) for ectopic pregnancy
	Ectopic pregnancy	Other causes	
PLTC	286 (91.7)	8359 (90.4)	1.16 (0.69–1.94)
MNM	25 (8.0)	745 (8.1)	0.99 (0.58–1.69)
MD	1 (0.3)	139 (1.5)	0.22 (0.05–1.01)
Total	**312**	**9243**	
Health indicators			LB: 82.144
MNMR	0.3/1000 LB	9.07/1000 LB	
SMOR	0.3/1000 LB	10.8/1000 LB	
MNM : MD ratio	25.0 : 1	5.4 : 1	
Mortality index	3.8%	15.7%	
MMR	1.2/100.000 LB	169.2/100.000 LB	

PR: prevalence ratio adjusted for cluster effect; PLTC: potentially life-threatening condition; MNM: maternal near miss; MD: maternal death; LB: live births; MNMR: maternal near miss ratio; SMOR: severe maternal outcome ratio; MMR: maternal mortality ratio.

**Table 2:** Prevalence of main causes of morbidity among cases complicated with ectopic pregnancy or other conditions and number of cases identified by specific WHO criteria for maternal near miss.

Causes of morbidity	Ectopic pregnancy	Other conditions	*P*
Hypertension	1.3	72.5	**<0.001**
Hemorrhage	94.6	21.5	**<0.001**
Infection	0.3	1.1	0.195
Clinical-surgical	4.5	10.9	**0.011**
Total	**(312)**	**(9,243)**	

WHO criteria for MNM and MD among ectopic pregnancy cases		MNM	MD
Clinical		(16)	(1)
Laboratory		(3)	(1)
Management		(15)	(1)
Total		25	1

MNM: maternal near miss; MD: maternal death. Values in bold type indicate statistically significant values.

**Table 3:** Estimated risk of ectopic pregnancy among maternal morbidity cases, according to conditions of severity management used.

Conditions of severity management	Ectopic pregnancy	Other conditions	PR for ectopic pregnancy	95% CI
Blood transfusion	37.2	15.7	**2.37**	**1.73–3.24**
Central venous access	2.6	3.8	0.67	0.33–1.35
ICU admission	7.7	22.6	**0.34**	**0.18–0.63**
Prolonged hospital stay (>7 days)	3.5	30.9	**0.11**	**0.05–0.25**
Nonanesthetic intubation	1.6	3.1	0.51	0.19–1.39
Return to operating room	1.6	3.4	0.48	0.14–1.64
Laparotomy	97.1	3.1	**31.39**	**21.87–45.04**
Use of magnesium sulphate	0.0	50.0	**<0.01**	**<0.01-<0.01**
Other major surgical procedures	1.0	0.8	1.27	0.30–5.31
Total	**(312)**	**(9,243)**		

PR: prevalence ratio adjusted for cluster effect; 95% CI: 95% confidence interval for prevalence ratio; ICU: intensive care unit. Values in bold type indicate statistically significant values.

**Table 4:** Estimated risk of worse outcome (MNM + MD) among maternal morbidity cases due to ectopic pregnancy according to some sociodemographic characteristics.

Characteristics	MNM + MD	PLTC	PR	95% CI
Age (years)				
10–19	11.5	6.6	1.83	0.78–4.27
20–29	46.2	52.1	(Ref.)	
30–39	34.6	36.0	1.08	0.37–3.15
40–49	7.7	5.2	1.58	0.31–8.08
(*n*)	(26)	(286)		
Marital status (a)				
Married/cohabitating	55.6	46.9	1.38	0.49–3.88
Single/separated/widow	44.4	53.1	(Ref.)	
(*n*)	(18)	(224)		
School education (b)				
Fundamental (primary)	47.1	42.8	(Ref.)	
Medium (high)	41.2	46.1	0.83	0.29–2.38
Superior (university)	11.8	11.2	0.96	0.17–5.34
(*n*)	(17)	(152)		
Skin color/ethnicity (c)				
White	72.7	37.5	(Ref.)	
Nonwhite	27.3	62.5	0.26	0.05–1.32
(*n*)	(22)	(192)		

MNM: maternal near miss; MD: maternal death; PLTC: potentially life-threatening condition; PR: prevalence ratio adjusted for cluster effect; 95% CI: 95% confidence interval for prevalence ratio. Missing values for (a) 70 cases; (b) 143; (c) 98.

**Table 5:** Estimated risk of worse outcome (MNM + MD) among maternal morbidity cases due to ectopic pregnancy according to some obstetric characteristics.

Characteristics	MNM + MD	PLTC	PR	95% CI
Previous abortions (a)				
None	95.7	66.8	(Ref.)	
1 or more	4.3	33.2	**0.10**	**0.01–0.78**
(*n*)	(23)	(271)		
Previous C-sections (b)				
None	86.4	76.9	(Ref.)	
1	9.1	18.1	0.47	0.11–2.01
2 or more	4.5	5.0	0.82	0.10–7.00
(*n*)	(22)	(260)		
Parity (c)				
0	37.5	35.8	(Ref.)	
1-2	45.8	48.3	0.91	0.34–2.45
≥3	16.7	15.9	1.00	0.29–3.52
(*n*)	(24)	(271)		
Previous uterine surgery (d)				
Yes	0.0	3.4	**<0.01**	**<0.01-<0.01**
No	100.0	96.6	(Ref.)	
(*n*)	(17)	(207)		
Gestational age at resolution (e)				
<9 weeks	71.4	73.2	(Ref.)	
≥9 weeks	28.6	26.8	1.09	0.32–3.64
(*n*)	(14)	(142)		
Substandard care—delays (f)				
Yes	22.7	15.0	1.59	0.55–4.57
No	77.3	85.0	(Ref.)	
(*n*)	(22)	(266)		

MNM: maternal near miss; MD: maternal death; PLTC: potentially life-threatening condition; PR: prevalence ratio adjusted for cluster effect; 95% CI: 95% confidence interval for prevalence ratio. Values in bold type indicate statistically significant values. Missing values for (a) 18 cases; (b) 30; (c) 17; (d) 88; (e) 156; and (f) 24 cases.

**Table 6:** Variables independently associated with a worse outcome (MNM + MD) among maternal morbidity cases due to ectopic pregnancy (*n* = 187).

Variable	Coefficient	SE coef.	*P*	PR_adj_ (95% CI)
Previous uterine scar	−22.65	0.71	** <0.001**	** <0.01** **(<0.01-<0.01)**
Skin color (nonwhite)	−1.84	0.87	**0.047**	** 0.16 (0.03–0.97**)
Constant	−1.74	0.38	** <0.001**	

MNM: maternal near miss; MD: maternal death.

PR_adj_: prevalence ratio adjusted for cluster effect and all remaining significant predictive factors.

Multiple Poisson regression, controlled by age (years); marital status (married/cohabitating: 1; others: 0); school education (up to high: 0; superior: 1); skin color (white: 0; nonwhite: 1); BMI (underweight/adequate: 0; overweight/obese: 1); previous abortion (0; ≥1 : 1); previous C-sections (0; ≥1: 1); parity (0; ≥1: 1); previous uterine scar (yes: 1; no: 0); gestational age at resolution (<9 weeks: 0; ≥9: 1); occurrence of substandard care delays (yes: 1; no: 0). Values in bold type indicate statistically significant values.
